# Preparation and characterization of polyethylene glycol/chitosan composite water-based wound healing lubricant

**DOI:** 10.3389/fbioe.2022.990249

**Published:** 2022-09-14

**Authors:** Li Gao, Xinyi He, Taohong Zhang, Peipei Li, Ruifang An

**Affiliations:** Department of Obstetrics and Gynecology, The First Affiliated Hospital of Xi’an Jiaotong University, Xi’an Jiaotong University, Xi’an, Shaanxi, China

**Keywords:** water-based lubricant, wound healing, polyethylene glycol (PEG), chitosan (CS), biocompatibility

## Abstract

The proportion of pregnant women giving birth through cesarean section is increasing annually worldwide. However, post-cesarean section scar diverticulum is a common condition that occurs and requires better surgical strategies than the methods currently used. We hypothesized that using biological lubricant topically on the incision area during C-section could be an option to minimize the scar. This water-based polyethylene glycol (PEG)/chitosan (CS) composite lubricant was prepared via ultrasonic blending. The product was characterized using scanning electron microscopy, X-ray photoelectron spectroscopy, X-ray diffraction, Fourier-transform infrared spectroscopy, and Raman spectroscopy. The thermal stability of the materials and their heat absorption and release during heating were analyzed using thermogravimetric analysis and differential scanning calorimetry. Tribological tests proved that the PEG/CS composite had a better lubrication effect than either the PEG or CS lubricant alone, and the cell viability experiments verified that it had good biocompatibility. Finally, application of the composite lubricant onto the backs of mice modeling full-thickness skin incisions further confirmed that the product improved both the re-epithelialization and the collagen levels of the wounded skin. In conclusion, we expect our newly formulated PEG/CS composite lubricant to be useful not only for managing post-cesarean section scar diverticulum but also for healing skin wounds in general.

## 1 Introduction

In the future, the world is expected to face a severe problem of “population aging,” caused mainly by the increase in average life expectancy due to improvements in medical treatments and the decrease in fertility rates stemming from the ever-increasing cost of living that renders child rearing financially prohibitive (Luo et al., 2021). Additionally, the expansion of the aging population could lead to labor shortages (Phiromswad et al., 2022), more environmental pollution (Zha et al., 2022), and an increased burden on medical care sector and old-age benefit dividends (Kollerup et al., 2022). At present, China is encountering a severe problem of population aging. To effectively solve this problem, the government has issued various policies to encourage childbirth, such as a three-child policy, extended maternity leave, and birth subsidies. Then, the development of obstetrics and gynecology services accelerated with the policy of encouraging childbirth. Although natural birth is still the most common form of delivery, increasingly more pregnant women are choosing painless birthing options, such as water delivery and cesarean section (Fobelets et al., 2019), especially those with a narrow pelvis, abnormal placenta, abnormal birth canal, premature water rupture, and abnormal fetus. However, post-cesarean section scar diverticulum (PCSD), which generally refers to lacunae formed as a result of poor healing of the uterine incision after the cesarean procedure (Zheng et al., 2020; Huang et al., 2022), is a common condition that can cause many problems in the new mother, such as chronic inflammation (Negro et al., 2017; Bi et al., 2021). Therefore, new medical strategies are required to effectively reduce the occurrence of PCSD.

At present, surgery is the main treatment option for this condition. (Zhou et al., 2018) found through clinical observations that vaginal repair surgery could restore the anatomical structure of patients with PCSD, allowing the women to obtain satisfactory obstetric results. Using the hysteroscopic electrocauterization method, (Dai et al., 2022) cauterized part of the endometrium to treat PCSD and found obvious repair effects, with the technique completely removing the scar from the previous cesarean section and the surrounding ectopic endometrium, thereby reducing the recurrence rate of the condition. However, although surgical treatment of PCSD is effective, it can also cause harm to the mother. Before cesarean section, a large amount of normal saline is usually applied to the wound site (the part cut with the scalpel). However, normal saline disinfects only the epidermis. Therefore, a useful strategy would be to replace normal saline with a liquid that can assist with wound repair and promote muscle growth. Water-based lubricants exhibiting good biocompatibility with the human body and a high lubrication effect have already been developed by some researchers (Qin et al., 2018; Lu et al., 2021). These are composed mainly of deionized water and lubricant additives, such as nanoparticles (Chen et al., 2021; Perumalsamy et al., 2021; Rahman et al., 2021; Sun et al., 2021; Tang et al., 2022), polymers (Liu et al., 2021; Perumalsamy et al., 2021; Wang et al., 2021; Zheng et al., 2021), and ionic liquids (Tang et al., 2018; Wang et al., 2018; Tang et al., 2019; Amann et al., 2020; Dong et al., 2020). Various types of additives are used for slightly different purposes. For example, nanoparticles and ionic liquids are commonly used in the mechanical field, whereas polymers are applied in the biomedical field owing to their good biocompatibility and antibacterial effects. Polyethylene glycol (PEG), a high-molecular-weight polymer that dissolves easily in water, exhibits good compatibility with many organic components as well as excellent lubricity, moisture retention capability, dispersibility, and adhesivity (Hu et al., 2021). Another polymer, soluble chitosan (CS), also displays excellent dispersibility in water, lubrication and antibacterial effects, and biocompatibility; therefore, it is also widely used in the biomedical field. For example, (Lu et al., 2017) prepared a sol using CS and PEG, which have good biocompatibility and applied it to the human body, revealing that it has an excellent lubrication effect under a high load.

In this study, we prepared a new type of composite lubricant by combining PEG and CS under ultrasonic conditions. The surface micromorphology of the composite lubricant was examined using scanning electron microscopy (SEM), and the materials were also characterized using X-ray photoelectron spectroscopy (XPS), X-ray diffraction (XRD), Fourier-transform infrared (FT-IR) spectroscopy, and Raman spectroscopy. The thermal stability of the materials and their heat absorption and release during heating were analyzed using thermogravimetric analysis (TGA) and differential scanning calorimetry (DSC). Tribological tests confirmed that the lubricant had a good lubrication effect and made cutting with the scalpel easier. Moreover, cell viability tests confirmed that the lubricant did not inhibit cell growth and demonstrated good biocompatibility, indicating that it could be applied to the human body. Additionally, a mouse model of a full-thickness skin incision created on the back of the animals was used to prove that the lubricant could accelerate wound healing. Our novel lubricant is expected to be widely used in future cesarean sections.

## 2 Materials and methods

### 2.1 Materials

PEG (average molecular weight: 3,500–4,500 Da; melting point: 54–58°C; pH: 4–7; chemical formula: HO(CH_2_CH_2_O)_n_H; elements: C, H, O) was purchased from Tianjin Damao Chemical Reagent Factory (Tianjin, China). CS (carboxymethyl carboxylation degree ≥80%; chemical formula: C_20_H_37_N_3_O_14_; elements: C, H, N, O) was purchased from Shanghai Lanji Biology (Shanghai, China). The reagents and chemicals used in this study were of analytical grade.

### 2.2 Sample preparation

PEG (3 g), CS (0.15 g), and deionized water (6.85 g) were weighed separately using an electronic balance and then mixed in a beaker. The mixture-containing beaker was then sealed with cling film and placed in an ultrasonic cleaner for 30 min of sonication, which finally yielded the water-based PEG/CS (30 wt%/1.5 wt%) composite lubricant (the PEG/CS lubricant).

### 2.3 Biocompatibility test

L929 cells at the logarithmic stage of growth were trypsinized, counted, and inoculated into a 96-well plate at a density of 1 × 10^4^ cells/well. Six wells were allocated for each of the following four groups: control, PEG, CS, and PEG/CS. The cells were cultured at 37°C under 5% CO_2_ and 95% humidity for 12 h, by which time they had adhered stably to the plate walls. The following treatments were then carried out: For the PEG/CS group, 10 μL of the fabricated PEG/CS lubricant was added; for the PEG group, 10 μL of 30 wt% PEG was added; for the CS group, 10 μL of 1.5 wt% CS was added; and for the control group, 10 μL of phosphate-buffered saline (PBS) was added. The 96-well plate was maintained under the same culture conditions as before. After 24 h, the plate was placed on an ultra-clean table and 10 µL of Cell Counting Kit-8 reagent was added to each well. Then, after incubation at 37°C for 1 h, the absorbance of the cells in each well was measured at 450 nm with a microplate reader (Bio-Rad, Hercules, CA, United States) and the number of viable cells was counted. The test was repeated three times for each group to ensure the accuracy of the experiment.

### 2.4 Wound-healing effect test

All animal experiments were approved by the Animal Care and Experiment Committee of the Medical College of Xi’an Jiaotong University. Eight-week-old male Balb/c mice (purchased from the Medical Experimental Animal Center of Xi’an Jiaotong University) weighing 20 ± 0.4 g each were randomly divided into a control group and a PEG/CS group (3 mice in each group). All mice were raised under specific pathogen-free conditions before and after the operation. The animals were first anesthetized with isoflurane. Then, under a sterile environment, an equal volume of the PEG/CS lubricant or PBS was applied to the intended incision site of the skin on the backs of the mice in the respective groups. Subsequently, a 10-mm-long full-thickness skin incision was created with a scalpel. The wound was rinsed with sterile saline and covered with a skin patch (3M Health Care). On the 14th day, the mice were euthanized by cervical dislocation under anesthesia, and a 1 cm^2^ patch of skin was excised using sterile instruments. The skin sample was fixed with paraformaldehyde for 24 h and then embedded in paraffin blocks for the generation of paraffin tissue sections for hematoxylin and eosin (H&E), Masson and CD31 immunofluorescence staining. The test was repeated three times for each group to ensure the accuracy of the experiment.

### 2.5 Tribological test

The lubricity of the fabricated product was tested using a reciprocating friction tester (GSR-2; Shenzhen, China). Ultra-high-molecular-weight polyethylene (UHMWPE) balls and 304 stainless steel disks were used as the friction-making materials, the relevant parameters of which are shown in Table 1. The balls and disks were wiped with acetone before use. The experiment was carried out at an ambient temperature, with a load of 10 N, a speed of 2 cm/s, and an experimental time of 30 min. The results for each group are an average of three replicate tests.

**TABLE 1:** Properties of the tribological test materials.

Material	Diameter (mm)	Density (g × cm−3)	Hardness (HV)	Tensile strength (MPa)
304 stainless steel disk	Φ30	7.93	≤210	520
UHMWPE ball	Φ9.525	0.93	35	40

### 2.6 Characterization of the lubricants

The surface micromorphology of the PEG/CS lubricant was observed using SEM (OXFORD Instruments, Oxfordshire, United Kingdom). XRD analysis (D8 Advance Diffractometer; Bruker, Rheinstetten, Germany) was carried out using Cu-Kα radiation (*λ* = 0.15406 nm), with the system run at 40 kV and 40 mA, and the crystal phase structure of the prepared samples was 5–50°. The chemical composition of the prepared samples was analyzed using XPS (Escalab 250Xi; Thermo Fisher Scientific, Waltham, MA, United States) at 0–1,300 eV, and the multi-peak fitting of the C and O elements was carried out using XPSpeak41 software. The corresponding functional groups were determined by comparing the binding energies of C and O. The chemical functional groups of the prepared samples were analyzed using FT-IR spectroscopy (Nicolet iS50 spectrometer, spectral resolution of 4 cm^−1^; Thermo Fisher Scientific) in the range of 500–4,000 cm^−1^. The interaction between materials was analyzed in the range of 100–3,500 cm^−1^ using Raman spectroscopy (Hr800; Horiba Jobin Yvon, Edison, NJ, United States) at an excitation wavelength of 532 nm. The thermal stability of the prepared samples was analyzed from ambient temperature to 1,000°C using TGA (NETZSCH Group, Selb, Germany). DSC was used to analyze heat absorption and release by the prepared samples during heating from ambient temperature to 1,000°C.

## 3 Results and discussion

### 3.1 SEM analysis

**FIGURE 1:**
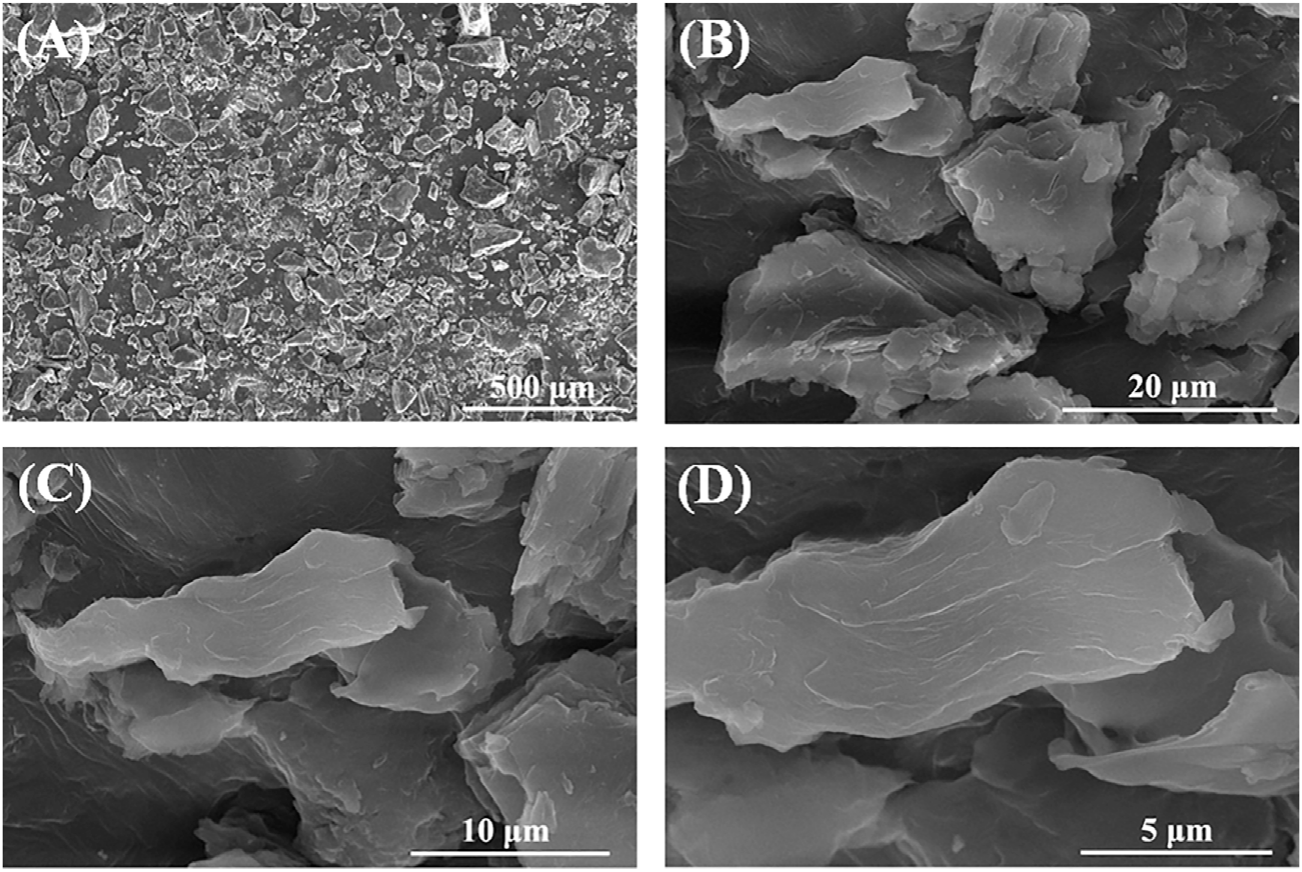
SEM images of PEG/CS composite lubricant at different scales: **(A)** 500 μm, **(B)** 20 μm, **(C)** 10 μm, and **(D)** 5 μm.

Figure 1 presents the SEM images of the PEG/CS lubricant at different scales. As seen in Figure 1A, the dried PEG/CS composite particles are lumpy in structure. Further enlargement of the image (Figure 1B) revealed that the massive particles were formed by the stacking of a large number of sheets. The edge of the flake is very obvious in the further enlarged images (Figures 1C,D). Thus, the dried PEG/CS lubricant was confirmed to be mainly flaky in structure, with the flakes appearing wrinkled. The spherical shape of CS could not be seen in the SEM images, likely because of its low content in the PEG/CS lubricant (Melo et al., 2020).

### 3.2 XPS and XRD analyses

**FIGURE 2:**
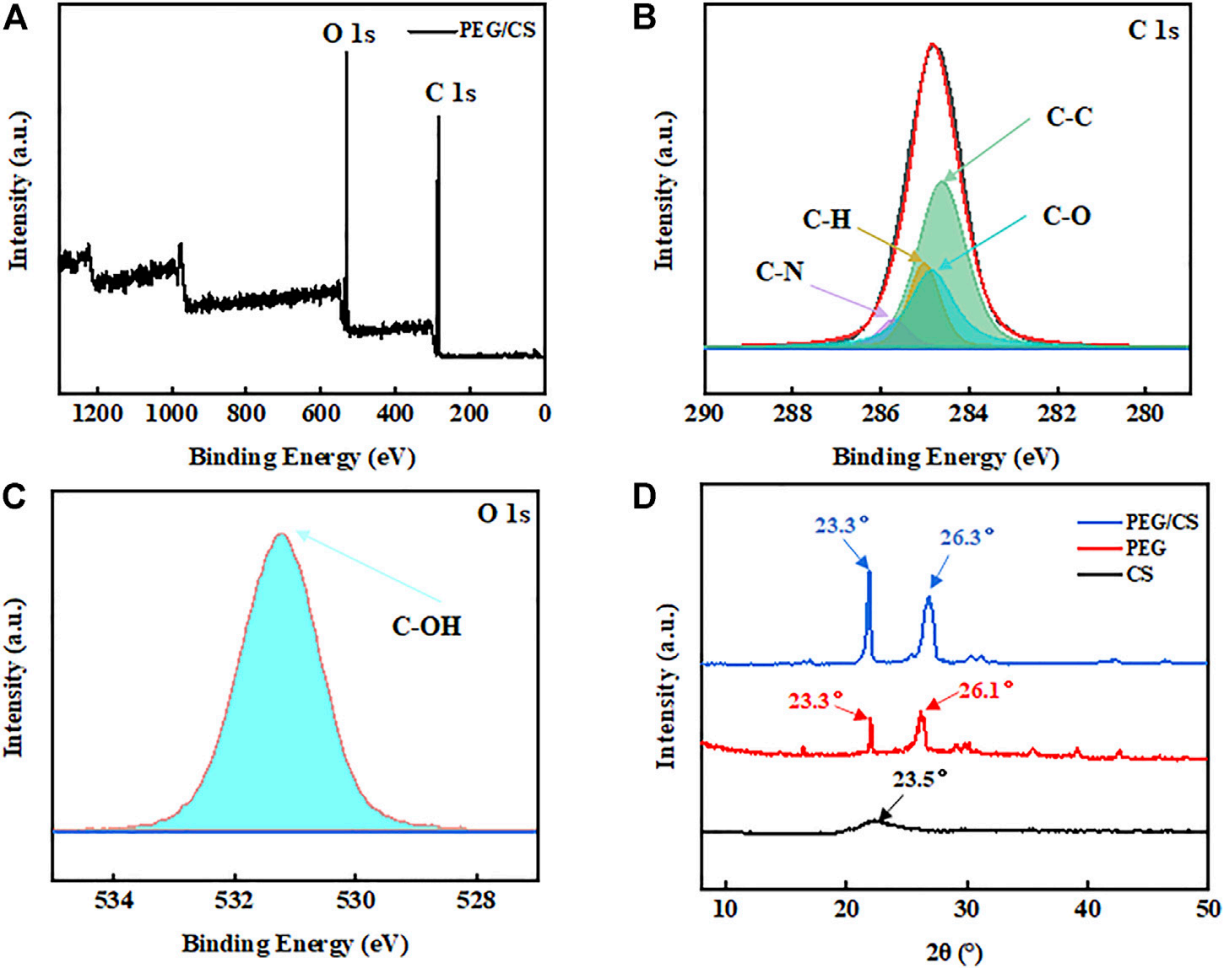
X-ray photoelectron spectroscopy (XPS) spectra of the PEG/CS composite lubricant: **(A)** Survey, **(B)** C 1s, **(C)** O 1s. **(D)** X-ray diffraction patterns of PEG, CS, and the PEG/CS composite.

The XPS spectrum of the PEG/CS lubricant and the fitted graphs corresponding to C 1s and O 1s are shown in Figures 2A,C. There were two high-intensity peaks in the spectrum for the PEG/CS lubricant; C 1s at 285.09 eV and O 1s at 531.7 eV. This proved that the PEG/CS lubricant was composed mainly of C and O elements. Further analysis of the two peaks using XPSpeak41 software revealed that the element C was mainly present in the form of C-C, C-O, C-H, and C-N (Figure 2B). C-C, C-O, and C-H were present in large quantities in both the PEG and CS monomers, whereas C-N was mainly present in CS. The element O was mainly present in the form of C-OH (Figure 2C) (Xu et al., 2016; Ren et al., 2019; Zhang et al., 2021). Thus, these results confirmed the presence of both PEG and CS in the fabricated lubricant.

Figure 2D shows the XRD signals of CS, PEG, and the PEG/CS composite. CS had a low and wide diffraction peak at 23.5°, indicating the low crystallinity of the sample. By contrast, PEG showed two relatively high and narrow diffraction peaks of higher intensity at 23.3° and 26.1° (Ren et al., 2019), indicating its higher crystallinity relative to that of CS. The composite sample also showed two relatively high and narrow diffraction peaks at 23.3° and 26.3°, and it can be seen that the intensity of the peak at 23.3° was enhanced, which may be related to the addition of CS. The PEG/CS composite mainly showed the diffraction peaks of the PEG sample, which may be related to the high content of this polymer. Moreover, the addition of a small amount of CS did not cause a great impact on the crystallinity of PEG.

### 3.3 Raman spectroscopy, FT-IR spectroscopy, TGA, and DSC analyses

**FIGURE 3:**
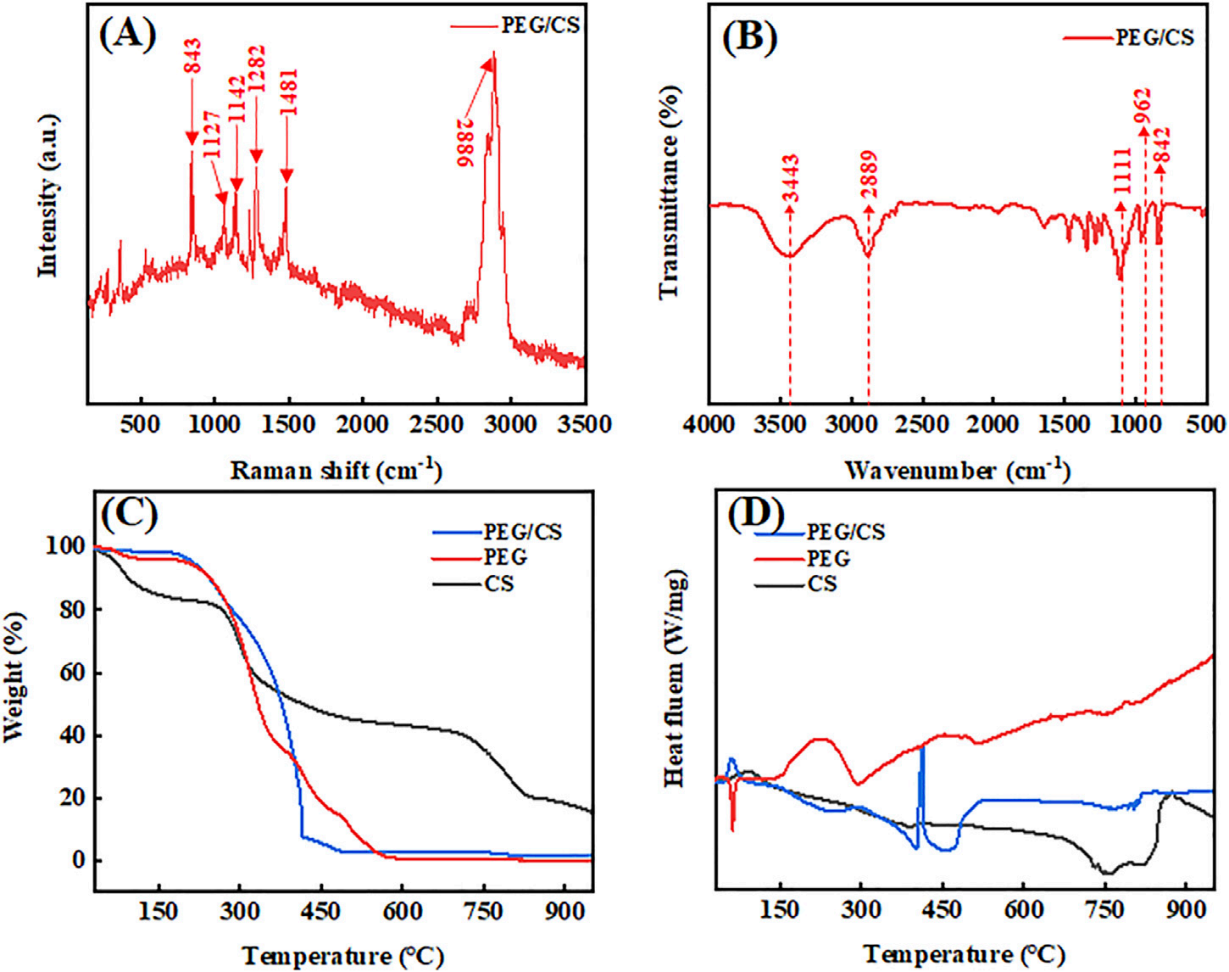
Results of the **(A)** Raman spectroscopy, **(B)** Fourier-transform infrared spectroscopy, **(C)** thermogravimetric analysis, and **(D)** differential scanning calorimetry analysis of the PEG/CS composite lubricant.

A Raman spectrum usually indicates the interactions between the materials of a substance. Figure 3A shows the Raman spectrum of the PEG/CS lubricant. According to a previous study, it can be inferred that the characteristic Raman bands at 843, 1,127, 1,142, 1,282, 1,481, and 2,886 cm^−1^ are mainly those of PEG (Lu et al., 2017), and the characteristic Raman bands at the other positions are similar to those of CS, proving that the PEG and CS molecules were well cross-linked by hydrogen bonds.

FT-IR imaging is an important method for detecting chemical functional groups. Figure 3B shows the FT-IR spectrum of the PEG/CS lubricant. The absorption bands at 3,443, 2,889, and 1,111 cm^−1^ correspond to the terminal hydroxyl group, the extension of the C-H bond in CH_2_, and the C-O-C functional group, respectively (Lu et al., 2017; Ren et al., 2019). These three characteristic absorption bands and monomers can be inferred from previous studies. The characteristic absorption bands at 962 and 842 cm^−1^ indicate the existence of C-C-O bonds, which are mainly due to PEG in the complex. It can be inferred from the above results that the chemical functional groups of PEG and CS did not change after ultrasonic mixing and that both polymers may be connected by hydrogen bonding, which is consistent with the results shown in Figure 3A.

TGA is usually applied to confirm the thermal stability of a sample. Figure 3C shows the TGA curves of PEG, CS, and the PEG/CS composite. The PEG sample showed a small mass loss at 80–150°C, which was mainly due to the small amount of water in the air and a large number of oxygen-containing functional groups in the molecule. By contrast, the sample showed a large mass loss at 250–550°C, which was due to degradation of the PEG backbone (Chen et al., 2021). The CS sample showed a small mass loss at 50–300°C, the reasons for which are similar to those for the PEG sample. It was apparent that CS had a higher mass than PEG from 300°C onward, which may indicate that CS has a better ability to bind water in the air, and residues still existed after heating to 950°C. The TGA curve of the PEG/CS sample was similar to that of PEG, which may be due to the high content of this polymer in the composite. Similar to the CS sample, there were still trace residues of the PEG/CS composite at 950°C (Gupta et al., 2009).

DSC typically indicates the amount of heat absorbed and released by a sample during heating. Figure 3D shows DSC traces for PEG, CS, and the PEG/CS composite. The PEG sample was endothermic at 75°C but showed an upward-trending peak at 450–600°C, indicating that it was exothermic. By contrast, the composite sample exhibited an exothermic reaction at 75°C. Comparison of the curves reveals that the peak position moved forward after the addition of CS, and the other positions also changed to different degrees. This may be due to the formation of a large number of hydrogen bonds between CS and PEG, which reduced the fluidity of the composite (Zhang et al., 2021).

### 3.4 Biocompatibility of the PEG/CS lubricant

**FIGURE 4:**
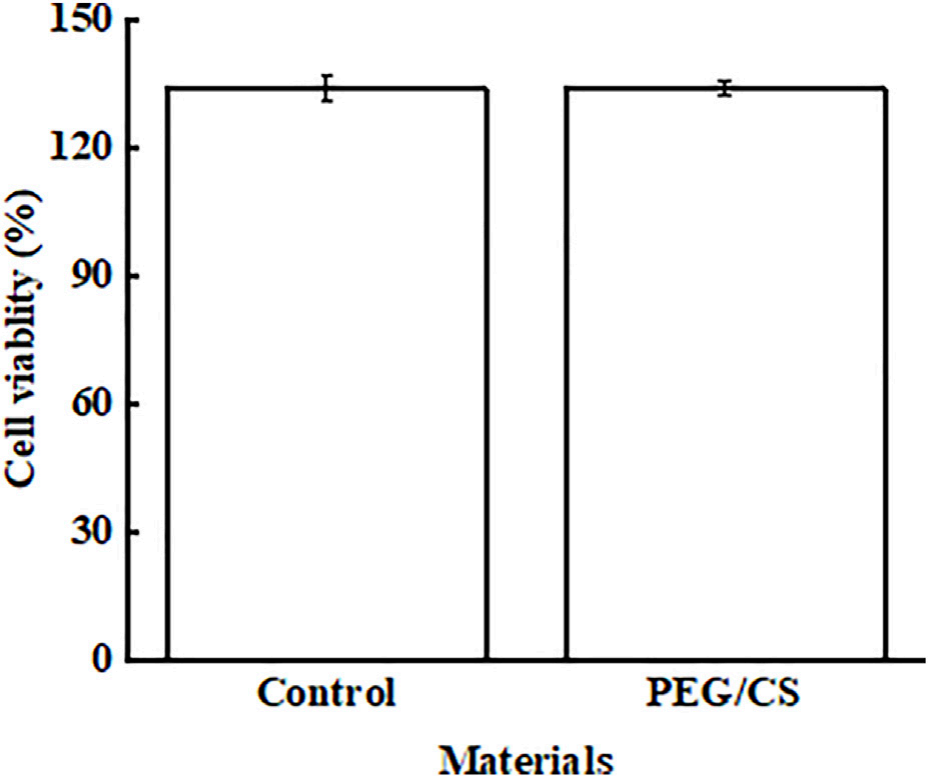
Viability of L929 cells treated with PBS (control) and PEG/CS, as determined with the Cell Counting Kit-8 assay.

Figure 4 shows the biocompatibility test results of the blank control group and PEG/CS lubricant. The survival rate of control L929 cells was 133.89%, which indicated that the cells proliferated in PBS buffer. Compared with the blank control L929 cells, the viability of cells treated with PEG/CS decreased slightly, and the cell survival rate was about 133.82%. However, the survival rate of the opposite cells decreased in value, but it also indicated that L929 cells proliferated in the presence of PEG/CS, which also proved that the polymer had good biocompatibility. (Lu et al., 2017; Hu et al., 2021).

### 3.5 Lubrication effects of the various polymers

#### 3.5.1 Lubrication affects PEG and CS alone

**FIGURE 5:**
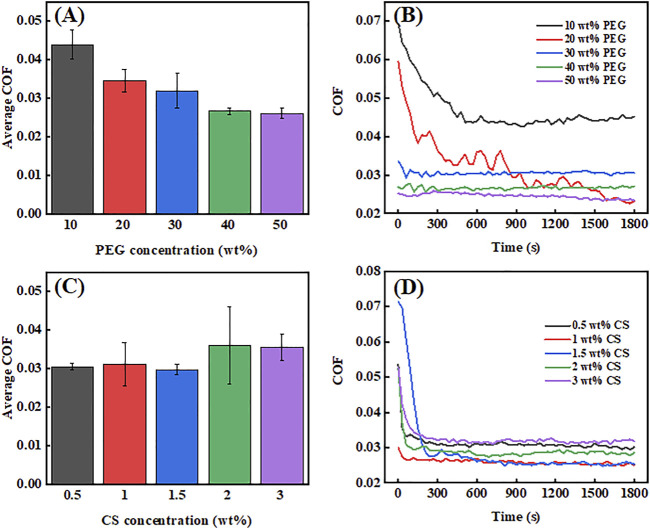
Average coefficient of friction (COF) values **(A)** and COF curves **(B)** of PEG at different concentrations. Average COF values **(C)** and COF curves **(D)** of CS at different concentrations.

In this experiment, the lubrication effects of PEG and CS individually, measured as the average coefficient of friction (COF), were investigated for different concentrations of each polymer. As evident in Figures 5A, 10 wt% PEG had an excellent lubrication effect, exhibiting a very low average COF of approximately 0.045. When the concentration of PEG was increased from 10% to 20 wt%, the average COF showed a faster reduction trend (Figure 5B). This was probably due to the increased concentration of the polymer and the greater contact area between its molecules and the friction contact area, resulting in more adequate lubrication. From the curves in Figure 5B, it can be seen that the COF values for the 10 wt% and 20 wt% PEG concentrations are high at the initial stage and then drop sharply within 10 min. This is because the film-forming characteristics of low-concentration PEG are poor, resulting in the peak COF values at the initial stage. With the increase in time, the UHMWPE balls start to wear out, resulting in wear debris that fills the uneven stainless steel disk and makes the contact surface smoother. With a further increase of the lubricant additive concentration to 50 wt%, the average COF showed a slow decline and dropped to a minimum of approximately 0.026, which is probably close to the optimum value for PEG lubrication. We speculate that with higher concentrations of PEG, the effect of the lubricant may barely change, and the average COF value may even increase. The reason for this is that the surface of PEG molecules contains a large number of oxygen-containing groups (e.g., -OH, -COOH, etc.), the high energies of which make them attract one another to form hydrogen bonds, resulting in the formation of large PEG agglomerates. It is difficult for such large agglomerated PEG molecules to enter the friction contact area, resulting in poor lubrication and serious wear on the contact surfaces of the friction-making pairs. Moreover, when the concentration of PEG is increased, the overall viscosity of the lubricant increases, resulting in a large viscous force in the friction contact area, which will have a significant impact on the situation where the average COF value is small; however, in this case, the contact surfaces of the friction-making pair will not be worn. As evident in Figure 5B, the higher the PEG concentration is, the lower the COF curves are over time, and the friction force is smaller. Moreover, the trends of four of the COF curves were relatively stable throughout the reciprocating process. Only the 20 wt% concentration showed slight changes in the average COF pattern over time, which may have been caused by other factors in the experimental process, but this had little influence on the average value of the overall curve.

Figure 5C shows the average COF values of CS at different concentrations. At the CS concentration of 0.5 wt%, the average COF value was already very low (∼0.03). It was also found that the average COF value changed only slightly between the CF concentrations of 0.5–1.5 wt%, dropping to a minimum of ∼0.028 at 15 wt% and then increasing at the concentrations of 2 and 3 wt%. However, the overall trend in average COF values was an increase rather than a decrease. The reasons for this phenomenon may be similar to those for PEG, in that the average COF value increases with the increase in lubrication additive concentration. First, agglomeration may occur, which prevents the lubrication additive from entering the friction contact area, resulting in an increase in the average COF value. Second, the increase in the concentration of the lubrication additive leads to increases in the viscosity of the lubricant, the viscosity of the contact surface, and thereby the average COF value. The COF curves in Figure 5D show the same trends as those in Figure 5C; that is, decreasing and then increasing with increasing CS concentration. All five friction curves showed a rapid decrease in the COF value during 300 s of reciprocating motion, followed by the maintenance of a relatively stable trend thereafter. This may be due to the initial stage of friction in the boundary lubrication. During the contact between the convex body and the small friction contact area, the friction caused by the reciprocating motion creates serious plastic deformation, which produces a large amount of friction debris that eventually fills the surface of the friction contact area. Therefore, after 300 s, the friction contact surface became smooth and flat because of the large amount of friction debris filling the substrate surface, which stabilized the COF curve.

#### 3.5.2 Lubrication effect of the PEG/CS lubricant

**FIGURE 6:**
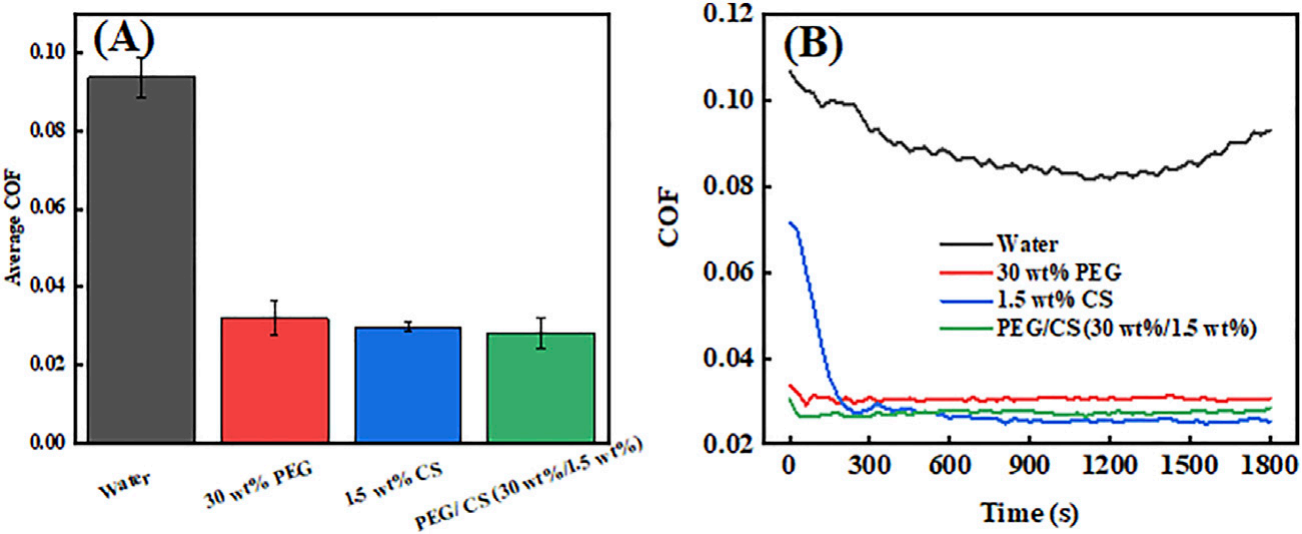
Average coefficient of friction (COF) values **(A)** and COF curves **(B)** of different lubricants.

The newly formulated PEG/CS lubricant was also subjected to tribological testing, and the results were compared with those of the single PEG and CS lubricants and water lubrication. Figure 6A shows the average COF images of four lubricants. The maximum average COF value with the water lubrication was approximately 0.09. By contrast, the single PEG and CS lubricants had much lower average COF values of approximately 0.032 and 0.028, respectively, which are approximately one-third that of the water lubrication result, indicating the excellent lubrication effect of the two single lubricants. However, the lubrication effect of the composite lubricant was the best, with an average COF value of approximately 0.027. This may be due to the synergistic lubrication effects of PEG and CS caused by the hydrogen bonds connecting them in the composite (which was confirmed by the Raman and FT-IR spectroscopy results). Figure 6B shows the changes in the COF curves of the different lubricants over time. For the water lubrication test, it can be seen that the curve is very high in the graph and the COF values are very large. With the increase in time, the COF values decreased gradually to the 1,200 s time point and then increased gradually again from 1,200 to 1,800 s, proving that the long-term lubrication effect of water cannot be maintained. However, for the other three lubricants, the COF values initially decreased and then tended to stabilize throughout the friction process. There were no significant abrupt changes in the curves during the 280–1,800 s period of the friction process, which proved that the three lubricants could maintain a good lubrication effect for a long time.

**FIGURE 7:**
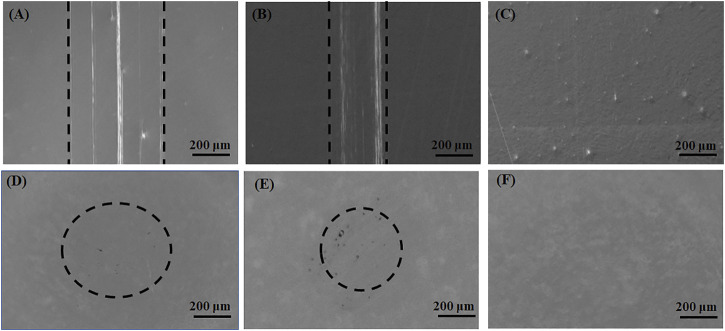
Optical microscopy images of the surfaces of stainless steel disks after tribological testing: **(A)** 30 wt% PEG, **(B)** 1.5 wt% CS, **(C)** PEG/CS (30 wt%/1.5 wt%). Optical microscopy images of the surfaces of ultra-high-molecular-weight polyethylene balls after tribological testing: **(D)** 30 wt% PEG, **(E)** 1.5 wt% CS, **(F)** PEG/CS (30 wt%/1.5 wt%).

Figure 7 shows the surface morphologies of the UHMWPE balls and the stainless steel disk wear in the tests using 30 wt% PEG (Figures 7A,D), 1.5 wt% CS (Figures 7B,E), and PEG/CS (30 wt%/1.5 wt%) lubricants (Figures 7C,F). Slight scratches were evident on the disk surface in the single PEG and CS lubrication tests, but almost no scratches appeared on the disk surface under PEG/CS lubrication, and the same situation occurred on the corresponding ball surface. It was confirmed that the composite lubricant was better than the single lubricants at protecting surfaces from frictional wear.

In summary, the PEG/CS lubricant maintained excellent lubrication for a long period of time during the tribological test, effectively reducing the shear force between the scalpel and skin during scalpel cutting, resulting in minimal damage at the skin incision, as confirmed by the later experiments on skin incision wound healing in a mouse model.

### 3.6 Wound-healing effect of the PEG/CS lubricant

#### 3.6.1 H&E

**FIGURE 8:**
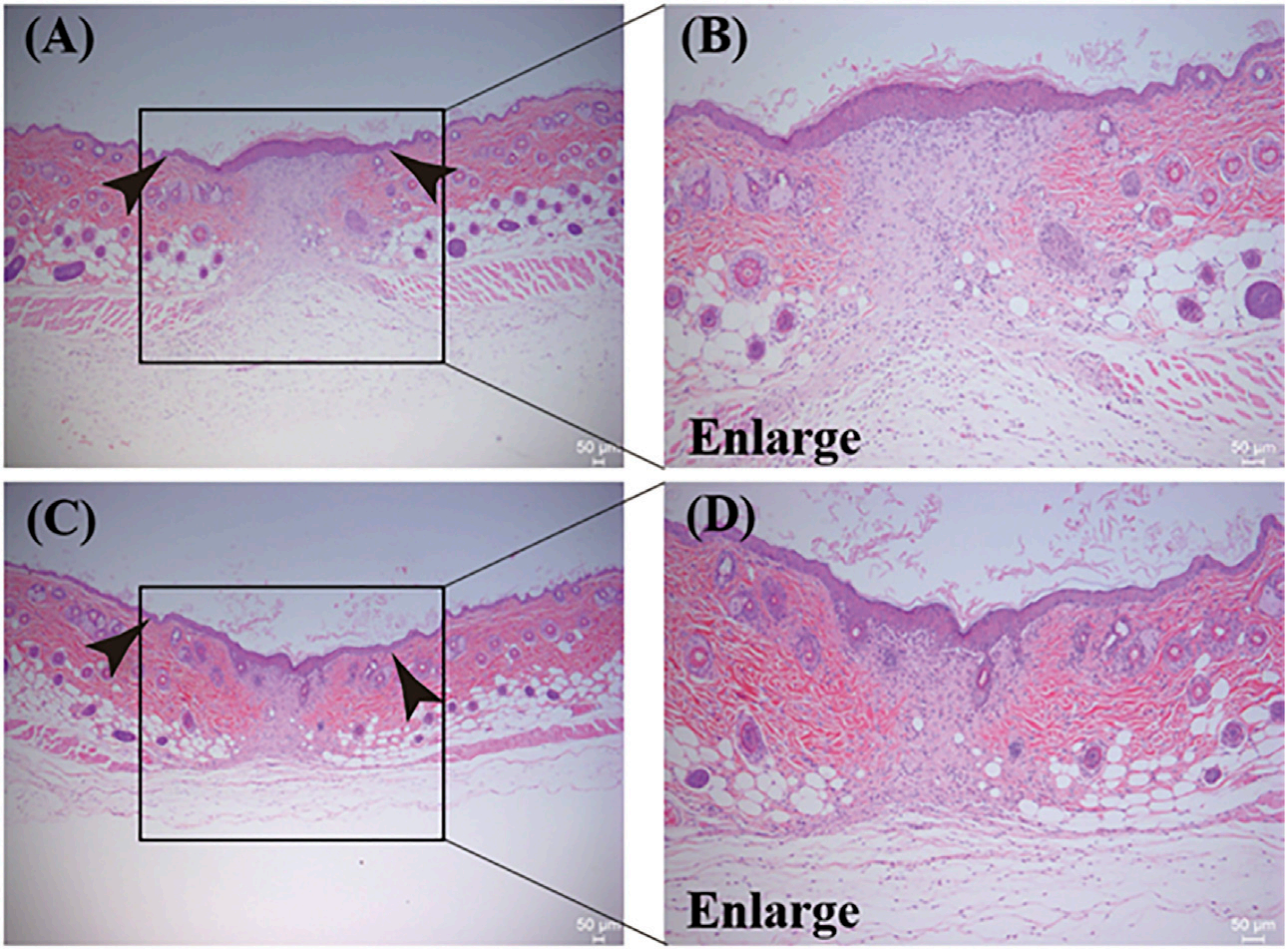
H&E staining results of mouse dorsal skin tissue protected by **(A)** PBS and **(C)** the water-based PEG/CS composite lubricant. **(B)** and **(D)** are magnified images of the corresponding boxed areas.

A very important process in skin wound healing is skin re-epithelialization, the level of which directly affects the wound-healing rate (Chen et al., 2018; Leng et al., 2020). In this experiment, the level of skin wound re-epithelialization was investigated using H&E staining of mouse tissue. Figures 8A,C show the H&E staining results for the PBS group and the PEG/CS lubricant group, respectively, whereas Figures 8B,D show magnified images of the boxed areas. A large number of inflammatory cells were evident in the PBS group, whereas there were significantly fewer inflammatory cells in the PEG/CS lubricant group. This may be due to the significant anti-inflammatory effect of CS in the PEG/CS composite (Chen et al., 2018). However, the arrangement of fibroblasts in both groups was regular, and there was a clear boundary between the dermis and epidermis and a clear cuticle on the edge of the epidermis. In summary, the wound tissue of the PEG/CS lubricant group showed better re-epithelialization and less scar formation, results that were supported by the quantitative analysis of the scar width (described below).

#### 3.6.2 Masson

**FIGURE 9:**
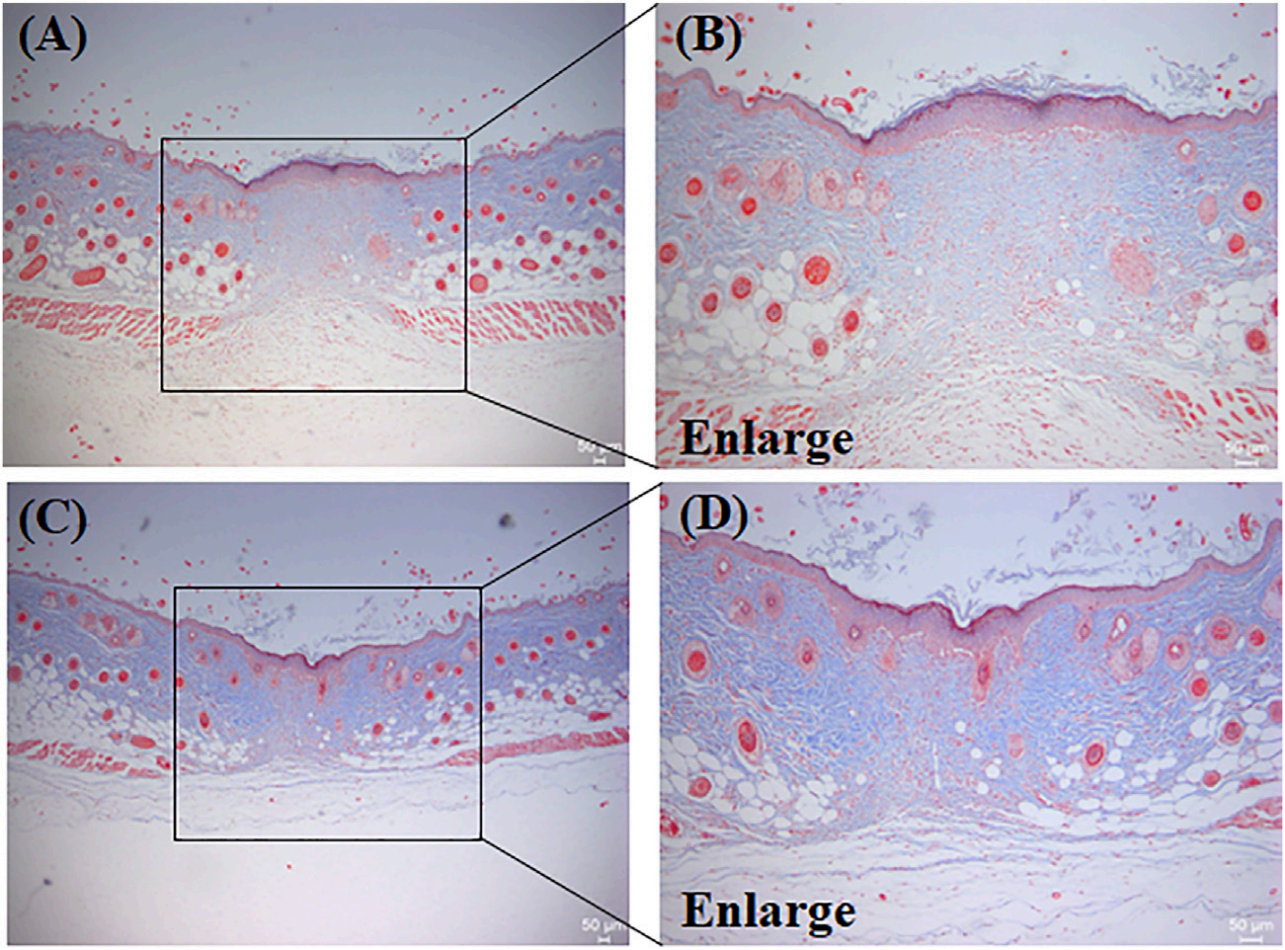
Masson staining results of mouse dorsal skin tissue protected by **(A)** PBS and **(C)** the water-based PEG/CS composite lubricant. **(B)** and **(D)** are magnified images of the corresponding boxed areas.

Collagen synthesis and collagen level are important factors in the healing of skin wounds (Chen et al., 2018; Leng et al., 2020). Masson staining was used to evaluate the collagen levels in the skin wounds of the PBS (Figures 9A,B) and PEG/CS lubricant groups (Figures 9C,D). The PEG/CS lubricant group had significantly more new collagen fibers in the skin wound than the PBS group. This may be because the PEG/CS lubricant promotes the growth of collagen fibrils. In summary, the tissue wound in the PEG/CS lubricant group exhibited a higher level of extracellular matrix deposition than that in the PBS group, and a large number of thick and tightly arranged collagen fibrils were observed.

#### 3.6.3 Summary of wound healing effect

**FIGURE 10:**
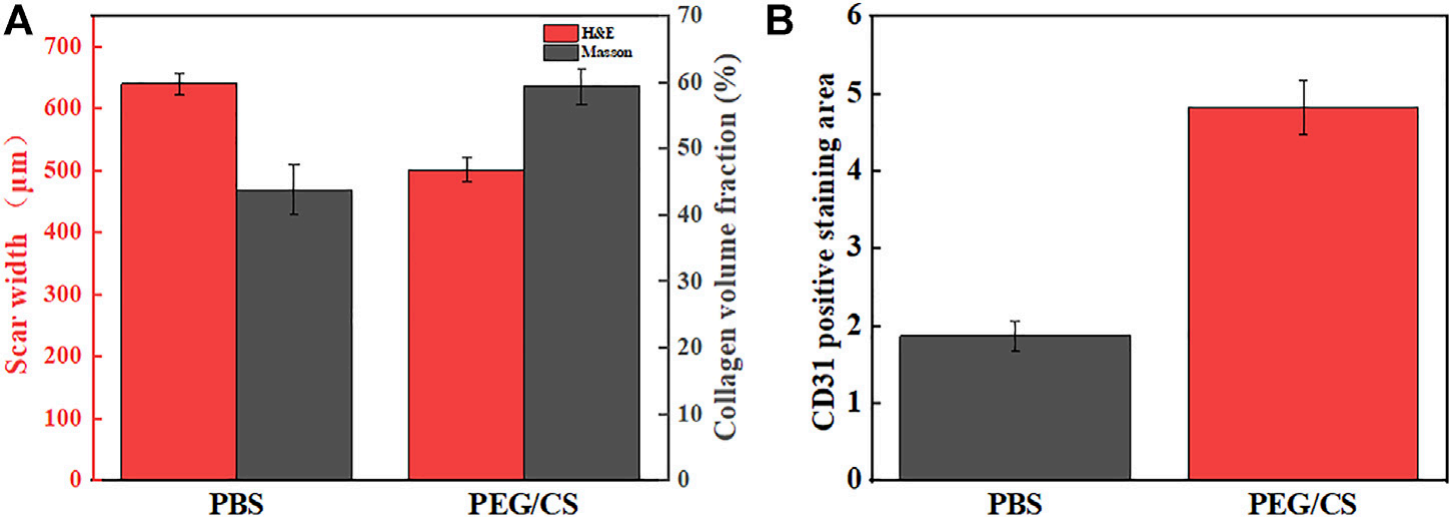
**(A)** Quantitative results of the H&E-stained scar width and Masson staining for collagen volume fraction, **(B)** statistical image of CD31 positive staining area.

Figure 10A shows the results of the quantitative analyses of the H&E-stained scar width and Masson staining for the collagen volume fraction. The PBS group had a scar width of 640 μm and a collagen volume fraction of 43.8%, whereas the PEG/CS lubricant group had a scar width of 501 μm and a collagen volume fraction of 59.3%. The PEG/CS group had a 139 μm reduction in the scar width and a 15.5% increase in the collagen volume fraction compared with the PBS group, confirming that it can accelerate the healing of skin wounds. Figure 10B shows the statistical image of CD31 positive staining area. It can be clearly seen from the image that the area of staining area in PEG/CS group is larger, about 2.5 times that of PBS group, which fully proves that the healing level of PEG/CS group is better.

### 3.7 Analysis of the wound-healing mechanism

**FIGURE 11:**
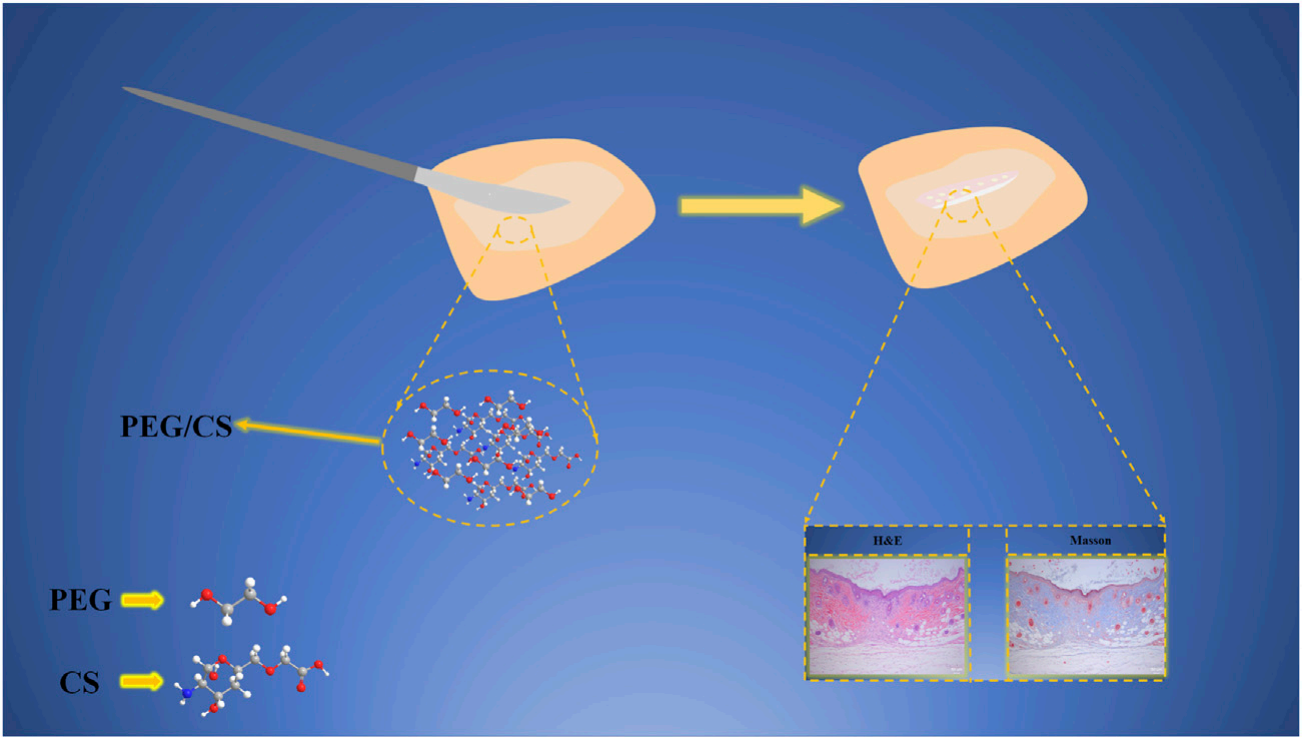
Wound-healing mechanism of the water-based PEG/CS composite lubricant.

Figure 11 shows the wound-healing mechanism of the PEG/CS lubricant. First, the lubricant forms a dense protective film on the skin surface. Thus, the shear force between the scalpel and the skin is decreased, which can effectively reduce skin damage during the cutting process. Additionally, the PEG and CS molecules in the composite lubricant are connected by hydrogen bonds, forming a dense network structure on the skin incision surface, which effectively isolates external pollutants. At this time, the lubricant compound quickly enters the wound, where the CS component accelerates wound healing mainly by promoting fibroblast proliferation and improving macrophage phagocytosis (Zhang et al., 2016; Yuan et al., 2018) while the PEG component promotes granulation tissue growth (Goh et al., 2016). Therefore, the PEG and CS molecules connected by hydrogen bonds in the composite can act synergistically to accelerate wound healing. Histological analyses by H&E and Masson staining verified that the application of the composite lubricant on the skin incision significantly reduced the inflammatory cell numbers and the scar size and significantly improved the re-epithelialization and collagen levels of the skin cells, confirming that the PEG/CS lubricant can effectively accelerate wound healing.

## 4 Conclusion

In this study, a water-based PEG/CS composite lubricant was successfully applied for wound healing. First, the PEG/CS lubricant was prepared via ultrasonic dispersion. Second, the samples were characterized by SEM, XRD, XPS, Raman spectroscopy, and FT-IR spectroscopy. The thermal stability of the sample was analyzed by TGA and its heat absorption and release were analyzed by DSC. The cell viability test verified that the PEG/CS lubricant had good biocompatibility, and the tribological test showed that it had a good lubrication effect. Finally, by establishing a full-thickness skin incision model on the backs of mice, and through H&E, Masson and CD31 immunofluorescence staining of the resultant skin tissue, we proved that the PEG/CS lubricant has a good wound-healing effect. We expect the PEG/CS lubricant to be applicable clinically.

## Data Availability

The raw data supporting the conclusion of this article will be made available by the authors, without undue reservation.

## References

[B1] Amann T. Chen W. Baur M. Kailer A. Ruehe J. (2020). Development of galvanically coupled plain bearings to reduce friction and wear. Forsch. Ingenieurwes. 84, 315–322. 10.1007/s10010-020-00416-z

[B2] Bi B. Gao S. Ruan F. Shi Y. Jiang Y. Liu S. (2021). Analysis on clinical association of uterine scar diverticulum with subsequent infertility in patients underwent cesarean section. Med. Baltim. 100, e27531. 10.1097/MD.0000000000027531 PMC8519233 34731147

[B3] Chen L. Tu N. Wei Q. Liu T. Li C. Wang W. (2021). Inhibition of cold-welding and adhesive wear occurring on surface of the 6061 aluminum alloy by graphene oxide/polyethylene glycol composite water-based lubricant. Surf. Interface Anal. 54, 218–230. 10.1002/sia.7044

[B4] Chen X. Cao X. Jiang H. Che X. Xu X. Ma B. (2018). SIKVAV-modified chitosan hydrogel as a skin substitutes for wound closure in mice. Molecules 23, 2611. 10.3390/molecules23102611 30314388 PMC6222830

[B5] Dai L. Wang H. Xing X. Peng Y. Wang Q. Li Q. (2022). An analysis of curative effect of combined transvaginal and hysteroscopic electrocauterization of partial endometrium to treat previous cesarean scar diverticulum. Minerva Surg. 77, 139–146. 10.23736/s2724-5691.21.08999-1 34342398

[B6] Dong R. Yu Q. Bai Y. Wu Y. Ma Z. Zhang J. (2020). Towards superior lubricity and anticorrosion performances of proton-type ionic liquids additives for water-based lubricating fluids. Chem. Eng. J. 383, 123201. 10.1016/j.cej.2019.123201

[B7] Fobelets M. Beeckman K. Buyl R. Healy P. Grylka-Baeschlin S. Nicoletti J. (2019). Preference of birth mode and postnatal health related quality of life after one previous caesarean section in three European countries. Midwifery 79, 102536. 10.1016/j.midw.2019.102536 31561129

[B8] Goh M. Hwang Y. Tae G. (2016). Epidermal growth factor loaded heparin-based hydrogel sheet for skin wound healing. Carbohydr. Polym. 147, 251–260. 10.1016/j.carbpol.2016.03.072 27178931

[B9] Gupta B. Arora A. Saxena S. Alam M. S. (2009). Preparation of chitosan–polyethylene glycol coated cotton membranes for wound dressings: Preparation and characterization. Polym. Adv. Technol. 20, 58–65. 10.1002/pat.1280

[B10] Hu F. Lu H. Ye Z. Zhang S. Wang W. Gao L. (2021). Slow-release lubrication of artificial joints using self-healing polyvinyl alcohol/polyethylene glycol/graphene oxide hydrogel. J. Mech. Behav. Biomed. Mat. 124, 104807. 10.1016/j.jmbbm.2021.104807 34492404

[B11] Huang L. Huang S. Yuan Y. Li Y. Chen M. Zhou C. (2022). Reduced pregnancy and live birth rates after *in vitro* fertilization in women with cesarean section scar diverticulum: A retrospective cohort study. J. Obstet. Gynaecol. Res. 48, 146–154. 10.1111/jog.15061 34734456

[B12] Kollerup A. Kjellberg J. Ibsen R. (2022). Ageing and health care expenditures: The importance of age per se, steepening of the individual-level expenditure curve, and the role of morbidity. Eur. J. Health Econ. 23, 1121–1149. 10.1007/s10198-021-01413-x 35037122

[B13] Leng Q. Li Y. Pang X. Wang B. Wu Z. Lu Y. (2020). Curcumin nanoparticles incorporated in PVA/collagen composite films promote wound healing. Drug Deliv. (Lond). 27, 1676–1685. 10.1080/10717544.2020.1853280 PMC7875550 33251864

[B14] Liu C. Yin Q. Li X. Hao L. Zhang W. Bao Y. (2021). A waterborne polyurethane-based leather finishing agent with excellent room temperature self-healing properties and wear-resistance. Adv. Compos. Hybrid. Mat. 4, 138–149. 10.1007/s42114-021-00206-3

[B15] Lu H. Chen L. Liu Q. Li Y. Gao L. (2021). Tribological properties of biocompatible molybdenum selenide nanoparticles as water lubrication additives for ultra-high molecular weight polyethylene/304 stainless steel contact. Mat. Chem. Phys. 272, 125053. 10.1016/j.matchemphys.2021.125053

[B16] Lu H. Ren S. Guo J. Li Y. Li J. Dong G. (2017). Laser textured Co-Cr-Mo alloy stored chitosan/poly(ethylene glycol) composite applied on artificial joints lubrication. Mater. Sci. Eng. C 78, 239–245. 10.1016/j.msec.2017.03.195 28575980

[B17] Luo Y. Su B. Zheng X. (2021). Trends and challenges for population and health during population aging — China, 2015–2050. China CDC Wkly. 3, 593–598. 10.46234/ccdcw2021.158 34594944 PMC8393078

[B18] Melo M. N. Pereira F. M. Rocha M. A. Ribeiro J. G. Diz F. M. Monteiro W. F. (2020). Immobilization and characterization of horseradish peroxidase into chitosan and chitosan/PEG nanoparticles: A comparative study. Process Biochem. 98, 160–171. 10.1016/j.procbio.2020.08.007

[B19] Negro S. Boutsikou T. Briana D. D. Tataranno M. L. Longini M. Proietti F. (2017). Maternal obesity and perinatal oxidative stress: The strength of the association. J. Biol. Regul. Homeost. Agents 31, 221–227. 28337896

[B20] Perumalsamy J. Gupta P. Sangwai J. S. (2021). Performance evaluation of esters and graphene nanoparticles as an additives on the rheological and lubrication properties of water-based drilling mud. J. Pet. Sci. Eng. 204, 108680. 10.1016/j.petrol.2021.108680

[B21] Phiromswad P. Srivannaboon S. Sarajoti P. (2022). The interaction effects of automation and population aging on labor market. PLoS One 17, e0263704. 10.1371/journal.pone.0263704 35134092 PMC8824351

[B22] Qin L. Feng X. Hafezi M. Zhang Y. Guo J. Dong G. (2018). Investigating the tribological and biological performance of covalently grafted chitosan coatings on Co-Cr-Mo alloy. Tribol. Int. 127, 302–312. 10.1016/j.triboint.2018.06.018

[B23] Rahman M. H. Warneke H. Webbert H. Rodriguez J. Austin E. Tokunaga K. (2021). Water-based lubricants: Development, properties, and performances. Lubricants 9, 73. 10.3390/lubricants9080073

[B24] Ren S. Lv L. Ma J. Lu H. Guo J. Li X. (2019). Slow-release lubrication effect of graphene oxide/poly(ethylene glycol) wrapped in chitosan/sodium glycerophosphate hydrogel applied on artificial joints. Mater. Sci. Eng. C 98, 452–460. 10.1016/j.msec.2018.12.109 30813047

[B25] Sun J. Meng Y. Zhang B. (2021). Tribological behaviors and lubrication mechanism of water-based MoO_3_ nanofluid during cold rolling process. J. Manuf. Process. 61, 518–526. 10.1016/j.jmapro.2020.11.044

[B26] Tang L. Zhang Y. Li C. Zhou Z. Nie X. Chen Y. (2022). Biological stability of water-based cutting fluids: Progress and application. Chin. J. Mech. Eng. 35, 3. 10.1186/s10033-021-00667-z

[B27] Tang W. Huang Z. Wang B. (2018). Synthesis of ionic liquid functionalized graphene oxides and their tribological property under water lubrication. Fullerenes Nanotub. Carbon Nanostructures 26, 175–183. 10.1080/1536383X.2017.1422246

[B28] Tang W. Wang B. Li J. Li Y. Zhang Y. Quan H. (2019). Facile pyrolysis synthesis of ionic liquid capped carbon dots and subsequent application as the water-based lubricant additives. J. Mat. Sci. 54, 1171–1183. 10.1007/s10853-018-2877-0

[B29] Wang L. Tieu A. K. Zhu H. Deng G. Cui S. Zhu Q. (2021). A study of water-based lubricant with a mixture of polyphosphate and nano-TiO_2_ as additives for hot rolling process. Wear 477, 203895. 10.1016/j.wear.2021.203895

[B30] Wang Y. Yu Q. Cai M. Zhou F. Liu W. (2018). Halide-free PN ionic liquids surfactants as additives for enhancing tribological performance of water-based liquid. Tribol. Int. 128, 190–196. 10.1016/j.triboint.2018.07.018

[B31] Xu X.-L. Zhou G.-Q. Li X.-J. Zhuang X.-P. Wang W. Cai Z.-J. (2016). Solution blowing of chitosan/PLA/PEG hydrogel nanofibers for wound dressing. Fibers Polym. 17, 205–211. 10.1007/s12221-016-5800-9

[B32] Yuan T. T. Foushee A. M. D. Johnson M. C. Jockheck-Clark A. R. Stahl J. M. (2018). Development of electrospun chitosan-polyethylene oxide/fibrinogen biocomposite for potential wound healing applications. Nanoscale Res. Lett. 13, 88. 10.1186/s11671-018-2491-8 29611009 PMC5880797

[B33] Zha D. Liu P. Shi H. (2022). Does population aging aggravate air pollution in China? Mitig. Adapt. Strateg. Glob. Chang. 27, 15. 10.1007/s11027-021-09993-y

[B34] Zhang W. Wang Y. Sui X. Sun Y. Chen D. (2016). Effects of chitin and sepia ink hybrid sponge on the healing of burning wound rats and its impact on macrophages *in vitro* . Acta Cir. Bras. 31, 119–125. 10.1590/S0102-865020160020000006 26959621

[B35] Zhang Z. Ye Z. Hu F. Wang W. Zhang S. Gao L. (2021). Double‐network polyvinyl alcohol composite hydrogel with self‐healing and low friction. J. Appl. Polym. Sci. 139, 51563. 10.1002/app.51563

[B36] Zheng F. Kong L. Wang H. Fan H. Gong H. Zhang K. (2020). Transvaginal three-dimensional ultrasound combined with HD flow model for uterus scar diverticulum. J. Infect. Public Health 13, 2014–2019. 10.1016/j.jiph.2019.06.030 31526641

[B37] Zheng Y. Asif A. Amiri A. Polycarpou A. A. (2021). Graphene-based aqueous drilling muds as efficient, durable, and environmentally friendly alternatives for oil-based muds. ACS Appl. Nano Mat. 4, 1243–1251. 10.1021/acsanm.0c02852

[B38] Zhou X. Yang X. Chen H. Fang X. Wang X. (2018). Obstetrical outcomes after vaginal repair of caesarean scar diverticula in reproductive-aged women. BMC Pregnancy Childbirth 18, 407. 10.1186/s12884-018-2015-7 30340551 PMC6194597

