# Corrigendum: Preparation and characterization of polyethylene glycol/chitosan composite water-based wound healing lubricant

**DOI:** 10.3389/fbioe.2023.1191529

**Published:** 2023-04-11

**Authors:** Li Gao, Xinyi He, Taohong Zhang, Peipei Li, Ruifang An

**Affiliations:** ^1^ Department of Obstetrics and Gynecology, The First Affiliated Hospital of Xi’an Jiaotong University, Xi’an Jiaotong University, Xi’an, Shaanxi, China

**Keywords:** water-based lubricant, wound healing, polyethylene glycol (PEG), chitosan (CS), biocompatibility

In the published article, there was an error in the author list, and authors Hailin Lu and Lu Chen were erroneously included and author Xinyi He was erroneously excluded. The corrected author list appears below.

“Li Gao, Xinyi He, Taohong Zhang, Peipei Li and Ruifang An*^”^

In the published article, there was an error in the author contributions, and authors HL and LC were erroneously included and author XH was erroneously excluded. The corrected author list appears below.

“LG and XH conceived the study and were in charge of overall direction and planning. XH, TZ, and PL performed the measurements; RA were involved in planning and supervising the work; and XH and LG processed the experimental data and performed the analysis. All authors discussed the results and commented on the manuscript.”

The authors apologize for these errors and state that this does not change the scientific conclusions of the article in any way. The original article has been updated.

